# Comparison of Three Non-Imaging Angle-Diversity Receivers as Input Sensors of Nodes for Indoor Infrared Wireless Sensor Networks: Theory and Simulation

**DOI:** 10.3390/s16071086

**Published:** 2016-07-14

**Authors:** Beatriz R. Mendoza, Silvestre Rodríguez, Rafael Pérez-Jiménez, Alejandro Ayala, Oswaldo González

**Affiliations:** 1 Departamento de Ingeniería Industrial, Universidad de La Laguna (ULL), 38203 La Laguna (Tenerife), Spain; bmendoza@ull.es (B.R.M.); aayala@ull.edu.es (A.A.); oghdez@ull.edu.es (O.G.); 2 Departamento de Señales y Comunicaciones, Universidad de Las Palmas de Gran Canaria (ULPGC), 35017 Las Palmas (Gran Canaria), Spain; rperez@dsc.ulpgc.es

**Keywords:** angle-diversity, sensor network, infrared channel, simulation, signal to noise ratio

## Abstract

In general, the use of angle-diversity receivers makes it possible to reduce the impact of ambient light noise, path loss and multipath distortion, in part by exploiting the fact that they often receive the desired signal from different directions. Angle-diversity detection can be performed using a composite receiver with multiple detector elements looking in different directions. These are called non-imaging angle-diversity receivers. In this paper, a comparison of three non-imaging angle-diversity receivers as input sensors of nodes for an indoor infrared (IR) wireless sensor network is presented. The receivers considered are the conventional angle-diversity receiver (CDR), the sectored angle-diversity receiver (SDR), and the self-orienting receiver (SOR), which have been proposed or studied by research groups in Spain. To this end, the effective signal-collection area of the three receivers is modelled and a Monte-Carlo-based ray-tracing algorithm is implemented which allows us to investigate the effect on the signal to noise ratio and main IR channel parameters, such as path loss and rms delay spread, of using the three receivers in conjunction with different combination techniques in IR links operating at low bit rates. Based on the results of the simulations, we show that the use of a conventional angle-diversity receiver in conjunction with the equal-gain combining technique provides the solution with the best signal to noise ratio, the lowest computational capacity and the lowest transmitted power requirements, which comprise the main limitations for sensor nodes in an indoor infrared wireless sensor network.

## 1. Introduction

Although most wireless sensor networks used are currently based on radio frequency (RF) systems [1,2], wireless optical communications are becoming an alternative to RF technology in some well-defined indoor application scenarios, such as environments where RF emissions are forbidden or restricted (health care, nuclear and chemical plants, etc.), video/audio transmission for in-home applications, secure network access or sensor networking [3]. Optical systems do not interfere with RF systems, thus avoiding electromagnetic compatibility restrictions. Moreover, there are no current legal restrictions involving bandwidth allocation and, since radiation is confined by walls, they produce intrinsically cellular networks, which are more secure against deliberate attempts to gain unauthorized access than radio systems. Despite the recent development in the field of visible light communications (VLC), the non-directed non-line-of-sight (non-LOS) infrared (IR) radiation has also been considered as a very attractive alternative to RF waves for indoor wireless local area networks, and therefore for indoor wireless sensor networking.

Sensor networks represent a significant improvement over traditional sensors. A sensor network can range from a few sensor nodes to a few hundred nodes capable of collecting data and routing them back to the sink. Data are routed back to the sink by a multihop infrastructureless architecture through the nodes. The sink node may communicate with the task manager, for example, via Internet. A sensor node consists of four basic components: a sensing unit, a processing unit, a transceiver unit, and a power unit, although they may also have additional application-dependent components such as a location finding system, power generator and mobilizer. In a sensor network, the sensor nodes are limited in power, computational capacities, and memory. Furthermore, the nodes should communicate untethered over short distances and mainly use broadcast communication. In a multihop sensor network, communicating nodes are linked by a wireless medium. As previously mentioned, most sensor networks are based on RF technology; however, another possible method for internode communication is by infrared, which enables the use of transceivers that are cheaper and easier to build. Current infrared-based sensor networks require a line-of-sight (LOS) between transmitter and receiver [1,2,3]. There are two basic classification schemes for wireless IR links [4]. In the first approach, the link can be directed by employing a narrow-beam transmitter and a narrow field of view (FOV) receiver, or non-directed, with a broad-beam transmitter and a wide FOV receiver. The second scheme classifies the links according to whether or not they rely on a line-of-sight (LOS) between the transmitter and the receiver (LOS and non-LOS configurations). The directed LOS IR method is the most efficient in terms of power consumption and can achieve very high bit rates. Its drawbacks are tight alignment requirement, immobility of the receiver, and interruptions in transmission caused by shadowing. These disadvantages are overcome in non-directed non-LOS methods (referred to as diffuse links), which utilize diffuse reflections from the ceiling and walls. These methods, however, suffer from ineffective power use and multipath dispersion, which tend to greatly limit the transmission rate. In an indoor wireless optical sensor network, it is not necessary to achieve high transmission rates, but it is necessary to limit power consumption, avoid blockage and shadowing, and reduce the impact of ambient light noise. In general, the use of angle-diversity detection makes it possible to reduce the impact of ambient light noise, path loss, and multipath distortion, in part by exploiting the fact that they often receive the desired signal received from different directions [4,5,6,7,8,9,10,11,12,13]. Angle-diversity detection can be implemented in two main ways: using a composite receiver with several branches looking in different directions (non-imaging angle-diversity receiver), or using an imaging receiver consisting of imaging optics and an array of photodetectors (imaging angle-diversity receiver).

The propagation characteristics of the indoor IR channel are fully described by the channel’s impulse response, which depends on multiple factors such as the room geometry, the reflection pattern of surfaces, the emitter and receiver characteristics and their relative locations. Indoor optical channel simulation can significantly enhance the study of optical wireless links. For this reason, in order to estimate the impulse response in IR wireless indoor channels, several simulation methods have been put forth [14,15], but all of them share the same problem, namely, the intensive computational effort. However, we make use of a Monte Carlo ray-tracing algorithm [16,17,18,19], which offers a lower computational cost than previous methods, especially when a high temporal resolution and a large number of reflections are required. In this paper, we study by simulation those indoor IR links that are characterised by the use of three non-imaging angle-diversity receivers as input sensor of nodes for an indoor IR wireless sensor network. The receivers, which have been proposed or studied by research groups in Spain, are the conventional angle-diversity receiver (CDR) [7,8,13], the sectored angle-diversity receiver (SDR) [11,12], and the self-orienting receiver (SOR) [20,21]. A conventional angle-diversity receiver uses multiple receiving elements or branches that are oriented in different directions, where each element employs its own filter and non-imaging concentrator, such as a compound parabolic concentrator (CPC) or hemispheric lens; a sectored receiver is a hemisphere where a set of parallels and meridians defines the photodetector boundaries; and finally, a self-oriented receiver is composed of a single-element detector that makes use of an optical front-end based on a lens, which is oriented towards the direction from which the highest signal to noise ratio (SNR) is received. The advantages achieved by angle-diversity reception depend on how the signals received in the different elements are processed and detected. When multipath distortion is significant, the optimum reception technique is maximum-likelihood combining (MLC), but its complexity is too high for many applications, and a number of simpler approaches are possible: maximal-ratio combining (MRC), selection best (SB), and equal-gain combining (EGC). When multipath distortion is negligible or it is not a significant parameter in the application scenario, such as in sensor networks, the optimum MLC reduces to MRC.

As noted earlier, current infrared-based sensor networks require a LOS link between transmitter and receiver. In this paper, we compare the use of SDR, CDR, and SOR as the input sensor for the nodes in an indoor IR wireless sensor network operating in a diffusive link for broadcast communication. This comparison is based on calculating the rms delay spread, the path loss, and the SNR when MRC, EGC, and SB combination techniques are employed. Based on the results of the simulations, we are going to demonstrate that a conventional angle-diversity receiver, used in conjunction with the equal-gain combining technique allows us to meet the requirements with the best SNR, the lowest computational capacity and the lowest transmitted power requirements. The use of angle-diversity receivers can be combined with multi-beam transmitters to significantly improve the power efficiency of diffuse links [4,10,22], which is desirable due to the power constraint of sensor nodes. This is an area for consideration in future research.

The remainder of the article is organized as follows: in Section 2, the channel model of the IR link for conventional, sectored, and self-orienting receivers using angle diversity is presented; i.e., the Monte Carlo ray-tracing algorithm used to study the signal propagation in the indoor IR channel and mathematical models employed to describe the effective signal-collection area of three receivers are described. Furthermore, we present the expressions for calculating the signal to noise ratio for an angle-diversity receiver as a function of the combining technique employed. Finally, in Section 3, we present several simulation results for comparing the performance of the use of three non-imaging angle-diversity receivers in IR links operating at low bit rates.

## 2. Channel Model and Signal-to-Noise Ratio

In optical wireless links, the most viable method is to employ intensity modulation (IM), in which the instantaneous power of the optical carrier is modulated by the signal. The receiver makes use of direct detection (DD), where a photodetector generates a current that is proportional to the instantaneous received optical power. The channel characteristics in an indoor optical wireless channel using IM/DD can be fully characterised by the impulse response *h(t)* of the channel [4]:

(1) I(t)=R x(t)⊗h(t)+n(t)

where *I(t)* represents the received instantaneous current at the output of the photodetector, t is the time, *x(t)* is the transmitted instantaneous optical power, ⨂ denotes convolution, *R* is the photodetector responsivity and *n(t)* is the background noise, which is modelled as white and Gaussian, and independent of the received signal.

### 2.1. Channel Impulse Response

To evaluate the impulse response of the indoor IR channel, a Monte Carlo ray-tracing algorithm was implemented. In general, the impulse response of the IR channel for arbitrary emitter *E* and receiver *R* positions can be expressed as an infinite sum of the form [14]:

(2) $$h(t;E,R)=h^{\left(0\right)}(t;E,R)+\sum_{k=1}^{\infty }h^{\left(k\right)}(t;E,R)$$

where *h^(0)^(t;E,R)* represents the LOS impulse response and *h^(k)^(t;E,R)* is the impulse response of the light undergoing *k* reflections, i.e., the multiple-bounce impulse responses.

**Figure 1:**
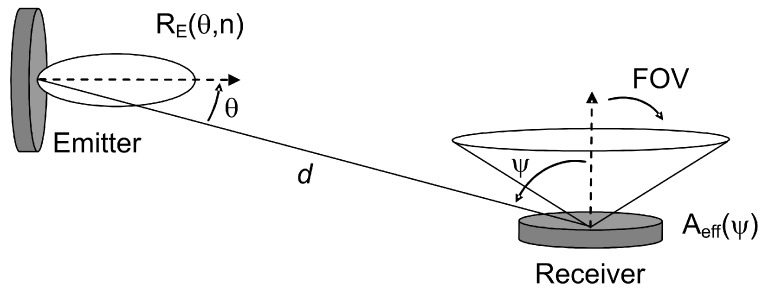
Emitter and receiver geometry without reflector.

Given an emitter *E* and receiver *R* in an environment free of reflectors (Figure 1), with a large distance d between both, the LOS impulse response is approximately:

(3) $$h^{\left(0\right)}(t;E,R)=\frac{1}{d^{2}}R_{E}(\theta ,n)A_{eff}(\psi )\delta \left(t-\frac{d}{c}\right)$$

where *R_E_(θ,n)* represents the generalized Lambertian model used to approximate the radiation pattern of the emitter, c is the speed of light and *A_eff_(ψ)* is the effective signal-collection area of the receiver [14].

In general, a bare detector, commonly called a single-element detector, achieves an effective signal-collection area of:

(4) $$A_{eff}^{bare}(\psi )=A_{R}cos(\psi )\;rect\left(\frac{\psi }{FOV}\right),\;where\;rect\left(x\right)=\{\begin{array}{cc} 1, & \left|x\right|\leq 1\\ 0, & \left|x\right|>1 \end{array}$$

where *A_R_* is the physical area of the receiver, and FOV is the receiver field of view (semi-angle from the surface normal).

**Figure 2:**
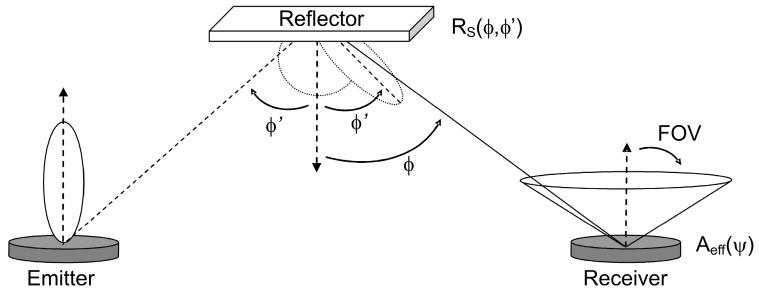
Emitter and receiver geometry with reflector. Reflection pattern of the surface is described by Phong’s model.

In an environment with reflectors, however, the radiation from the emitter can reach the receiver after any number of reflections (see Figure 2). In the algorithm, to calculate the impulse response due to multiple reflections, many rays are generated at the emitter position with a probability distribution equal to its radiation pattern.

The power of each generated ray is initially *P_E_/N*, where *N* is the number of rays used to discretize the optical source. When a ray impinges on a surface, the reflection point becomes a new optical source, thus a new ray is generated with a probability distribution provided by that surface’s reflection pattern. The process continues throughout the maximum simulation time, *t_max_*. After each reflection, the power of the ray is reduced by the reflection coefficient of the surface, and the reflected power reaching the receiver (*p_i,k_*, *i-*th ray, *k-*th time interval) is computed by:

(5) $$p_{i,k}=\frac{1}{d^{2}}R_{S}(\varphi ,\varphi^{\prime })A_{eff}(\psi )$$

where *R_S_(φ,φ′)* is the model used to describe the reflection pattern. In this work, Phong’s model is used [17]. In this model, the surface characteristics are defined by three parameters: the reflection coefficient *ρ*, the percentage of incident signal that is reflected diffusely *rd*, and the directivity of the specular component of the reflection *m*.

Therefore, the total received power in the *k-*th time interval (width ∆*t*) is calculated as the sum of the power of the *N_k_* rays that contribute in that interval:

(6) $$p_{k}=\sum_{i=1}^{N_{k}}p_{i,k}=\sum_{i=1}^{N_{k}}\frac{1}{d^{2}}R_{S}(\varphi ,\varphi^{\prime })A_{eff}(\psi )$$

Letting *M* = *t_max_/*∆*t*, and assuming as the time origin the arrival of the LOS component, the impulse response after multiple reflections is given by:

(7) $$\sum_{k=1}^{\infty }h^{\left(k\right)}(t;E,R)=\sum_{j=0}^{M-1}p_{k}\delta (t-j\Delta t)$$

Replacing Equations (3) and (7) in Equation (2), the channel impulse response can be expressed as:

(8) $$h(t;E,R)=\frac{1}{d^{2}}R_{E}(\theta ,n)A_{eff}(\psi )\delta (t)+\sum_{j=1}^{M-1}p_{k}\delta (t-j\Delta t)$$

Once the impulse response *h(t)* is computed for a fixed emitter *E* and receiver *R* position, the main parameters that characterise the IR channel—the path loss (*PL*) and the rms delay spread (*D*)—are easily calculated as [4]:

(9) PL=−10logH(0)   H(f)=∫−∞∞h(t)e−j2πtfdtD=[∫−∞∞(t−μ)2h2(t)dt∫−∞∞h2(t)dt]12 μ=∫−∞∞th2(t)dt∫−∞∞h2(t)dt

### 2.2. Effective Signal-Collection Area Model for Non-Imaging Angle-Diversity Receivers

In general, the use of angle-diversity receivers makes it possible to reduce the impact of ambient light noise, path loss and multipath distortion, in part by exploiting the fact that they often receive the desired signal from different directions. Another advantage of angle-diversity reception is that it allows the receiver to simultaneously achieve a high optical gain and a wide FOV. In this section we describe the models for the effective signal-collection area of three non-imaging angle-diversity receivers that have been proposed or analysed by research groups in Spain. The receivers are called the conventional angle-diversity receiver, the sectored angle-diversity receiver, and the self-orienting receiver.

#### 2.2.1. Conventional Angle-Diversity Receiver

**Figure 3:**
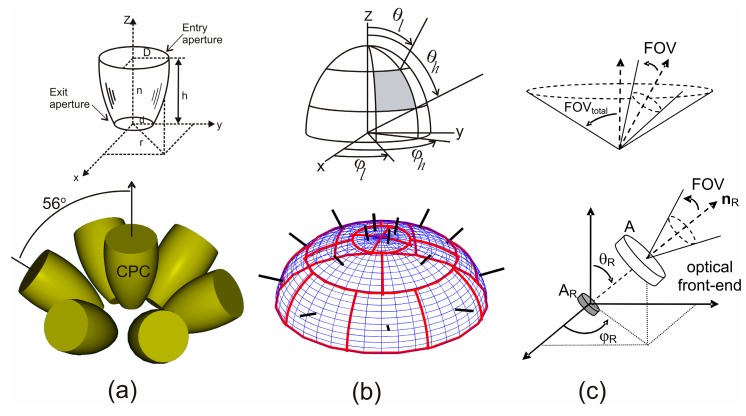
Non-imaging angle-diversity receiver geometries: (**a**) Conventional angle-diversity receiver; (**b**) Sectored angle-diversity receiver; (**c**) Self-orienting receiver.

A conventional angle-diversity receiver uses multiple receiving elements or branches that are oriented in different directions, where each element employs its own filter and non-imaging concentrator, such as a compound parabolic concentrator or hemispherical lens (see Figure 3a).

By adding a filter and concentrator to a bare detector, the effective signal–collection area of the receiver becomes:

(10) $$A_{eff}^{c,f}(\psi )=A_{R}T_{S}(\psi )g(\psi )cos(\psi )rect\left(\frac{\psi }{\pi /2}\right)$$

where *T_S_(ψ)* is the filter transmission and *g(ψ)* the concentrator gain. Non-imaging concentrators exhibit a trade-off between gain and FOV. An idealized non-imaging concentrator [4] having an internal refractive index *n* achieves a constant gain expressed as:

(11) $$g(\psi )=\frac{n^{2}}{{sin}^{2}\psi_{c}}rect\left(\frac{\psi }{\psi_{c}}\right)$$

where *ψ_c_* is the concentrator FOV, which is usually less than or equal to *π/*2. In the model used, the concentrator gain is affected by the optical efficiency *η(ψ)*, which represents the reflection losses of the concentrator. The propagation delay introduced by the concentrator is also considered [18]:

(12) $$g(\psi )=\frac{n^{2}}{{sin}^{2}\psi_{c}}\eta (\psi )rect\left(\frac{\psi }{\pi /2}\right),\;t(\psi )\neq 0$$

Replacing *g(ψ)* in the expression that defines the effective signal–collection area of the receiver yields:

(13) $$A_{eff}^{c,f}(\psi )=\frac{n^{2}A_{R}T_{S}(\psi )cos(\psi )}{{sin}^{2}\psi_{c}}\eta (\psi )rect\left(\frac{\psi }{\pi /2}\right)$$

In addition, in a wireless IR communications system, an optical bandpass filter can be used to limit the ambient radiation reaching the detector. A common form of bandpass filter consists of a stack of dielectric thin-film layers. By properly choosing the number of layers, their thicknesses, and their refractive indexes, it is possible to control the surface reflectance and thus the filter transmittance. The filter transmission *T_S_(ψ)* can be described fairly accurately by a simple five-parameter model [23]. In this model, for radiation of wavelength *λ_o_* incident at angle *ψ*, the filter transmission is given by:

(14) $$T(\psi ;\Delta \lambda ,\psi^{\prime })=\frac{T_{0}}{1+{\left[\frac{\lambda_{0}-\lambda^{\prime }(\psi ;\psi^{\prime })}{\Delta \lambda /2}\right]}^{2m}}$$

where *ψ′* is the filter orientation, *T_0_* is the peak transmission at *ψ′*, *∆λ* is the spectral half-power bandwidth, *m* is the filter order and *λ′(ψ;ψ′)* represents the shifting to shorter wavelengths at non-normal incidences, which is described by:

(15) λ′(ψ;ψ′)=λ0(ns2−n12sin2ψns2−n12sin2ψ′)12

where *n_1_* is the index of the input layer and n_s_ is the effective index for the spacer layer. The design of the optical filter thus boils down to specifying the two parameters *∆λ* and *ψ′*.

The remaining three parameters (*n_s_*, *m* and *T_0_*) should be chosen to be as large possible, while considering the constraints imposed by technology. In keeping with reference [23], we used *n_s_* = 2.293, *m* = 3, and *T_0_* = 0.92. Furthermore, to provide the best utilization of the CPC and filter, the angular bandwidth *∆ψ* should be equal to the concentrator FOV. Figure 3a shows a conventional angle-diversity receiver composed of seven elements, one of them oriented vertically towards the ceiling and six angled at a 56° elevation with a 60° separation in azimuth. Each element uses a bandpass optical filter and a CPC with a 50° field of view. The receiver structure resulted from a study focused on designing the conventional angle-diversity receiver that offers the best performance with respect to the path loss and the rms delay spread [13].

#### 2.2.2. Sectored Angle-Diversity Receiver

A sectored angle-diversity receiver consists of a set of photodetectors located on a hemisphere. The space of sectored receivers enclosed between two parallels is called a crown, and a sector is the region of the crown enclosed between two equally spaced meridians. Every sector in each crown has an equal azimuth aperture, and therefore the same limiting elevation angles. In summary, the sectored receiver is defined by a set of parameters [11], Ψ, which specifies, for each crown, its number of sectors, *N_S_*, the azimuth offset of its first sector, ε, and its limiting elevation angles, *θ_l_* and *θ_h_*. As an example, bottom illustration in Figure 3b shows the configuration of a sectored receiver with three crowns, which is defined by Ψ = {(4, 0°, 0°, 24°), (4, 20°, 24°, 53°), (8, 0°, 53°, 9°)}. As we can see, the first crown in the sectored receiver consists of 4 sectors (*N_S_* = 4). The azimuth offset of its first sector is ε = 0°, and its limiting elevation angles are 0° and 24° (*θ_l_* = 0° and *θ_h_* = 24°). Analogously to the conventional angle-diversity receiver shown in Figure 3a, the configuration of the sectored receiver was the result of a study focused on achieving the receiver structure that exhibits the minimum simultaneous rms delay spread and path loss [12].

As with a single-element detector, each sector of the sectored receiver is defined by its position, orientation, vertical and horizontal apertures, and effective signal-collection area. The region of a sector is specified by the two limiting elevation angles, *θ_l_* and *θ_h_*, and the two limiting azimuth angles, *ϕ_l_* and *ϕ_h_*, where *θ_l_ < θ_h_* and *ϕ_l_ < ϕh* (see top illustration in Figure 3b). Consequently, the vertical aperture is given by (*θ_h_ − θ_l_*)/2, whereas the horizontal aperture is set by (*ϕ_h_* − *ϕ_l_*)/2. The orientation of each sector is defined by its azimuth and elevation angles (*ϕ_S_,θ_S_*), which are given by:

(16) $$\phi_{S}=\left(\phi_{l}+\phi_{h}\right)/2\;and\;\theta_{S}=\left(\theta_{l}+\theta_{h}\right)/2$$

except in the case of a polar crown with a single sector where *θ_S_* = 0° (sector aimed vertically upward, *ϕ_h_ − ϕ_l_* = 2π, *θ*_h_ any, *θ_l_* = 0). Each sector in a sectored receiver has an effective signal-collection area given by:

(17) $$A_{eff}(\psi )=A_{R}T_{S}\left(\psi \right)cos(\psi )rect\left(\frac{\psi }{\pi /2}\right)$$

where *T_S_(ψ)* is the transmission characteristic of the filter used to limit the ambient radiation reaching each photodetector, *ψ* is the incidence angle of radiation with respect to the sector orientation, and *A_R_* represents the physical area of the detector, which is described by:

(18) $$A_{R}=r^{2}\cdot \left(\phi_{h}-\phi_{l}\right)\cdot \left(cos\;\theta_{l}-cos\;\theta_{h}\right)$$

where *r* is the radius of the hemisphere. In a sectored receiver, the optical filter should be deposited or bonded onto the outer surface of the sectored receiver, i.e., onto all the sectors or photodetectors that comprise the receiver. In this sense, a bandpass or interference optical filter cannot be used because the radiation that reaches each photodetector is incident upon the filter at any value of *ψ* between 0° and 90°, shifting the filter transmission to shorter wavelengths and minimizing its transmission. Therefore, the ambient light radiation and the desired signal striking the filter at wide incidence angles will not be adequately filtered. However, in order to reject ambient light, longpass absorption optical filters could be used [4], which pass light at every wavelength beyond the cutoff wavelength. They are usually made of coloured glass or plastic and their transmission characteristics are constant and substantially independent of the incidence angle, i.e., *T_S_(ψ)* = *T_0_* for all wavelengths longer than the cutoff wavelength. Since the silicon device does not respond to wavelengths beyond about 1100 nm, the filter-photodetector combination exhibits a bandpass optical response, whose bandwidth will range from the filter cutoff wavelength to the longest wavelength at which the silicon responds. Although the sectored receiver may currently be regarded as a theoretical structure since no prototype has yet been implemented, we assume that a longpass absorption filter with a 780-nm cutoff wavelength is employed, specifically the Schott RG-780 optical filter [24]. This filter exhibits an almost constant transmission characteristic above 780 nm given by *T_0_* = 0.99, and consequently, the spectral bandwidth resulting from the filter-photodetector combination is about *Δλ* = 320 nm.

#### 2.2.3. Self-Orienting Receiver

The third angle-diversity receiver considered is called a self-orienting receiver (see Figure 3c). The self-orienting receiver consists of a single-element detector which employs an optical front-end based on a lens [20,21]. The receiver is mounted on an electromechanical orienting system controlled by a digital signal processor (DSP). An SNR estimator and a maximum search algorithm, which is based on a modified version of Simulated Annealing (SA), are used to automatically aim the receiver at the area of the room with the highest SNR [20,21]. From the description above, we can deduce that the receiver is actually a modified version of a select-best angle diversity receiver, which allows operating with very narrow FOV. In general, the self-orienting receiver can be represented by the parameters *p_R_*, *n_R_*, FOV_total_, *A*, *A_R_*, and FOV, i.e., its position, orientation, total FOV, aperture area of the lens, physical area of photodetector and the FOV of the system formed by the detector and the optical front-end. Therefore, its effective signal-collection area can be expressed as:

(19) $$A_{eff}(\psi )=A_{R}T_{S}(\psi )g(\psi )cos(\psi )rect\left(\frac{\psi }{\pi /2}\right)$$

where *T_S_(ψ)* is the filter transmission and *g(ψ)* = *G* represents the optical front-end gain. Replacing the gain *G* by *A*/*A_R_* in the expression, and assuming an ideal filter, the expression that defines the effective signal-collection area of the receiver can be expressed as:

(20) $$A_{eff}(\psi )=A_{R}\;G\;cos(\psi )rect\left(\frac{\psi }{\pi /2}\right)=A\;cos(\psi )rect\left(\frac{\psi }{\pi /2}\right)$$

In keeping with the proposal made in [20,21], we have assumed that the self-orienting receiver uses a positive lens system with an aperture diameter of 1.5 cm as an optical concentrator, along with a circular photodetector with area *A_R_* = 3.53 mm^2^ and responsivity *R* = 0.6 A/W to collect the signals received. The concentrator provides a high optical gain (*G* = 50) at the expense of a narrow FOV, which is only 3°. This extremely narrow FOV allows us to use an ideal bandpass optical filter with a narrow spectral bandwidth (*Δλ* = 50 nm) for better optical noise rejection.

### 2.3. Signal to Noise Ratio

In general, the received signal-to-noise ratio can be expressed by:

(21) $$SNR={\left(R\;P_{S}\right)}^{2}/\sigma^{2}$$

where *P_S_* is the received optical signal average power, *R* is the photodetector responsivity and *σ^2^* represents the total noise variance [4,25]. The total noise variance can be calculated by the sum of the contributions from the background light-induced shot noise and thermal noise due to the amplifier:

(22) $$\sigma^{2}=\sigma_{shot}^{2}+\sigma_{thermal}^{2}$$

The shot noise variance can be approximated by:

(23) $$\sigma_{shot}^{2}\approx 2qRI_{2}R_{b}P_{bg}$$

where *q* = 1.6 × 10^−19^ C is the electron charge, *I_2_* = 0.562 is a noise bandwidth factor, *R_b_* is the bit rate, and *P_bg_* is the incident optical power from natural and artificial ambient light.

Assuming the use of a FET-based transimpedance preamplifier [4], the thermal noise variance can be expressed as:

(24) $$\sigma_{thermal}^{2}=\frac{4kT}{R_{f}}I_{2}R_{b}+\frac{16\pi^{2}kT}{g_{m}}\left(\Gamma +\frac{1}{g_{m}R_{D}}\right)C_{T}^{2}I_{3}R_{b}^{3}+\frac{4\pi^{2}KI_{D}^{a}C_{T}^{2}}{g_{m}^{2}}I_{f}R_{b}^{2}$$

where *k* is Boltzmann’s constant, *T* is absolute temperature, *R_f_* is the feedback resistor, *g_m_* is the transconductance, *C_T_* is the total input capacitance of receiver, *R_D_* is the polarisation resistance, *Γ* is the FET channel noise factor, *K* and *a* are the FET *1/f* noise coefficients, *I_D_* is the FET drain current, and *I_2_*, *I_3_* and *I_f_* are noise-bandwidth factors. Neglecting stray capacitance [10,22], the total input capacitance *C_T_* is given by *C_T_* = *C_d_* + *C_g_*, where *C_d_* and *C_g_* are the detector and FET gate capacitances, respectively. The detector capacitance *C_d_* is proportional to the detector area *A_R_*, i.e., *C_d_* = *η* *A_R_*, where *η* represents the fixed photodetector capacitance per unit area. Therefore, replacing *C_T_* by *η* *A_R_* + *C_g_* in Equation (24) yields:

(25) $$\sigma_{thermal}^{2}=\frac{4kT}{R_{f}}I_{2}R_{b}+\frac{16\pi^{2}kT}{g_{m}}\left(\Gamma +\frac{1}{g_{m}R_{D}}\right){\left(\eta A_{R}+C_{g}\right)}^{2}I_{3}R_{b}^{3}+\frac{4\pi^{2}KI_{D}^{a}{\left(\eta A_{R}+C_{g}\right)}^{2}}{g_{m}^{2}}I_{f}R_{b}^{2}$$

### 2.4. Signal to Noise Ratio for Angle-Diversity Receivers

Assuming no co-channel interference, a *J*-element angle-diversity receiver yields *J* receptions of the form:

(26) $$\begin{array}{cc} I_{j}(t)=R\;x(t)⊗h_{j}(t)+n_{j}(t), & j=1,\ldots ,J \end{array}$$

where *I_j_(t)* and *n_j_(t)* represent, respectively, the received instantaneous current and the total noise at the output of the *j*-th receiving element, *t* is the time, *x(t)* is the transmitted instantaneous optical power, *h_j_(t)* is the channel between the transmitter and the *j*-th receiver, ⨂ denotes convolution, and *R* is the responsivity of the *j*-th receiver, which are considered equal for all *J* receiving elements.

In general, much of the performance of an angle diversity receiver depends on how the signals received in the different elements *I*j*(t)* are processed and detected. There are several possible diversity schemes that can be considered. The most common techniques include MLC, MRC, SB, and (EGC) [4]. When multipath distortion is significant, the optimum reception technique is MLC, also known as matched-filter combining (MFC). In this technique, each *I_j_(t)* is processed by a separate continuous-time matched filter *h_j_(-t)* of the *j*-th receiving element. The output from each matched-filter is sampled and adjusted in magnitude by a weight factor *w_j_*, which is chosen to be inversely proportional to the power-spectral density (PSD) of the noise *n_j_(t)* at the output of the *j*-th receiving element. The weighted output signals are summed in order to obtain the combined signal. The main problem of MLC is its highly complex implementation, since this technique requires estimating each of the *J* channel impulse responses and noise PSD’s separately. For this reason, MLC is not suited to many applications. There are simple alternatives to MLC that are practical to implement, such as MRC, SB and EGC, which differ depending on how the signals are weighed and combined.

Independent of the combined method and of the weight factor, and according to Equation (21), the signal to noise ratio at the output of the *j*-th receiving element *SNR_j_*, and the SNR of the combined channel are defined as:

(27) $$SNR_{j}={\left(R\;P_{S,j}\right)}^{2}/\sigma_{j}^{2}$$

(28) $$SNR_{combined}=\frac{{\left(\sum_{j=1}^{J}w_{j}RP_{S,j}\right)}^{2}}{\sum_{j=1}^{J}w_{j}^{2}\sigma_{j}^{2}}$$

where *P_S,j_* and σ*^2^_j_* represent the received optical average power and the total noise variance of the *j*-th receiving element, respectively.

In MRC, the *I_j_(t)*, *j* = 1, …, *J*, are summed together with weights *w_j_* proportional to the signal current to noise-variance ratios, thereby maximizing the SNR of the weighted sum. Simulations have shown that MRC can reduce transmitter optical power requirements by 4–6 dB in diffuse links at low bit rates [26]. Moreover, in situations where the ambient noise and the strong signal components arrive from different directions, MRC can decrease the multipath distortion in comparison to a single, wide FOV receiver. When multipath distortion is not significant, the optimum MLC reduces to MRC. MRC requires estimating the SNR’s at the each of the receiving elements, thus increasing the complexity over non-diversity reception.

In order to obtain the weight factor *w_j_* at the *j*-th receiving element, the *J* partial-derivatives of the combined SNR with respect to each of the *w_j_*, *j* = 1, …, *J* are calculated and set equal to zero, so *w_j_* becomes:

(29) $$w_{j}=\frac{\sum_{j=1}^{J}w_{j}^{2}\sigma_{j}^{2}}{\sum_{j=1}^{J}w_{j}RP_{S,j}}\cdot \frac{RP_{S,j}}{\sigma_{j}^{2}}=k_{0}\cdot \frac{RP_{S,j}}{\sigma_{j}^{2}}$$

where *k_0_* is a constant independent of *j*, and *w_j_* is proportional to the signal current to noise-variance ratio, as expected.

Replacing Equation (29) in Equation (28), the SNR using MRC can be calculated as the sum of each SNR of the *j*-th receiving elements, which is given by:

(30) $$SNR_{MRC}=\frac{{\left(\sum_{j=1}^{J}\left(k_{0}\cdot \frac{RP_{S,j}}{\sigma_{j}^{2}}\right)RP_{S,j}\right)}^{2}}{\sum_{j=1}^{J}{\left(k_{0}\cdot \frac{RP_{S,j}}{\sigma_{j}^{2}}\right)}^{2}\sigma_{j}^{2}}=\sum_{j=1}^{J}\frac{{\left(RP_{S,j}\right)}^{2}}{\sigma_{j}^{2}}=\sum_{j=1}^{J}SNR_{j}$$

The SB method chooses the branch or receiving element with the highest SNR from among all branches. This technique can often improve SNR since it separates the signal from ambient light noise, but the gains are not as large as those achieved using MRC. Simulations have shown that SB requires 1–2 dB more transmitter optical power than MRC [27]. Also, in [11], it was shown that a reduction in multipath delay spread occurs when directional narrow FOV receivers are employed in the branches, making SB a suitable technique for high bit-rate systems. On the other hand, SB is not simpler to employ than MRC, since it requires estimating the SNR at each diversity branch. The SNR using SB is given by:

(31) $$\begin{array}{cc} SNR_{SB}=\underset{j}{max}\left(\frac{{\left(RP_{S,j}\right)}^{2}}{\sigma_{j}^{2}}\right), & 1\leq j\leq J \end{array}$$

EGC corresponds to MRC, but without attempting to weigh the signals; that is, the weights of all the received signals *I_j_(t)* are equal to a constant. This technique increases the receiver FOV but is unable to separate the signal from ambient noise. Moreover, using EGC can result in an increase in multipath distortion, making it unsuitable for very high bit rate links. The main advantage of EGC is that it avoids the need to estimate the SNR [7]. The SNR using EGC is not dependent on the constant weight value, and its expression is given by:

(32) $$SNR_{EGC}=\frac{{\left(\sum_{j=1}^{J}wRP_{S,j}\right)}^{2}}{\sum_{j=1}^{J}w^{2}\sigma_{j}^{2}}=\frac{{\left(\sum_{j=1}^{J}RP_{S,j}\right)}^{2}}{\sum_{j=1}^{J}\sigma_{j}^{2}}$$

## 3. Simulation Results and Discussion

**Figure 4:**
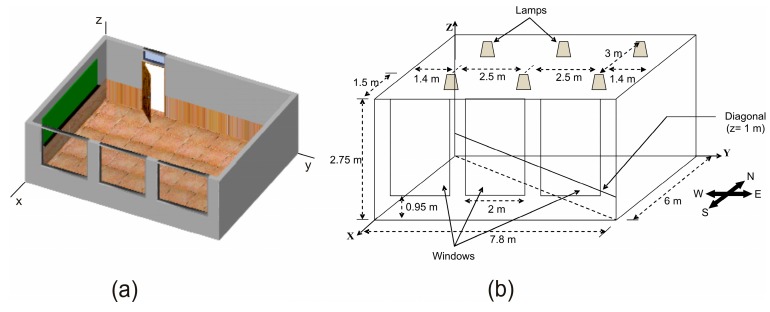
Graphical representation of the room: (**a**) 3D design; (**b**) Display of dimensions.

In this section, simulations are used to compare the performance of the three non-imaging angle-diversity receivers described in the previous section and shown in Figure 3: the conventional angle-diversity receiver (CDR), the sectored angle-diversity receiver (SDR), and the self-orienting receiver (SOR). The comparison is based on calculating the rms delay spread, the path loss, and the SNR when the MRC, SB, and EGC combination techniques are employed. To this end, the simulation algorithm described in the previous section was implemented, the models for the three non-imaging angle-diversity receivers were included, and the IR signal propagation for different configurations of optical links in the room shown in Figure 4 was studied. The simulation tool implemented allows us to study the infrared signal propagation inside any simulation environment or 3D scene. The tool features two fully differentiated parts. The first is charged with defining the 3D scene, which the user can describe by means of any CAD software that is capable of generating or storing the scene in 3DS format. In our simulations, we have used the Blender graphic design program because it offers multi-platform support in a freeware product that can output a 3DS file. The second part consists of implementing the propagation model. This refers to the mathematical models that characterize the effect of each of the elements present in the simulation environment (reflecting surfaces, emitters, and receivers), and to the simulation algorithm that, aided by these models, allows the channel response to be computed. The part of the tool that implements the propagation model and into which the 3D scene is input was programmed in C++. A detailed description of the simulation tool developed was presented in [19], where the parallelization of the simulation algorithm was also discussed.

The indoor environment shown in Figure 4 was based on the channel models for a 14 × 14 × 3 m^3^ office, a 6 × 6 × 3 m^3^ living room, and an 8 × 8 × 3 m^3^ hospital room, which were proposed by the IEEE802.15.7 VLC work group [28]. The room selected is almost analogous to the living room, but with a rectangular instead of square shape. Furthermore, in order to make the comparison as independent as possible of the characteristics of the emitter and the furniture distribution in the room, no obstacles and a diffuse emitter were assumed.

In general, the topology of an infrared-based sensor network can vary from a simple star network to a multihop wireless mesh network. However, regardless of the network topology, the sensor nodes should be able to collect data and route them to the sink node. That is, in a wireless sensor network there is a large number of tiny transmitters collecting data and routing them to a small number of receiver nodes. For this reason, in order to compare the performance of the three angle-diversity receivers used as the input sensor of a receiver sensor node, we have proposed a simulation scenario consisting of an angle-diversity receiver located in the centre of the room and multiple transmitters uniformly distributed along the diagonal from the northwest to the southeast of the room, as shown in Figure 4b. As previously mentioned, the angle-diversity receiver is located in the centre of the room, 1 m above the floor and aimed vertically towards the ceiling. We have assumed that the CDR uses photodetectors with a physical area of *A_R_* = 1 cm^2^, the SOR employs a photodetector with an area of 3.53 mm^2^, and the sectored receiver makes use of a hemisphere of radius *r* = 1.4 cm, meaning the largest sector has a physical area of about 1 cm^2^. Specifically, the sectors in the first, second and third crown of the sectored receiver have an area of 0.27 cm^2^, 0.95 cm^2^ and 0.92 cm^2^, respectively. Every photodetector in the three angle-diversity receivers has a responsivity of 0.6 A/W. As for the emitters, they are oriented vertically towards the ceiling, 1 m above the floor, located in thirty-seven locations uniformly distributed along the northwest-southeast diagonal of the room, and modelled as a first-order Lambertian emitter with a total emitted power of 15 mW. The remaining parameters used in the simulations match those shown in Table 1.

**Figure 5:**
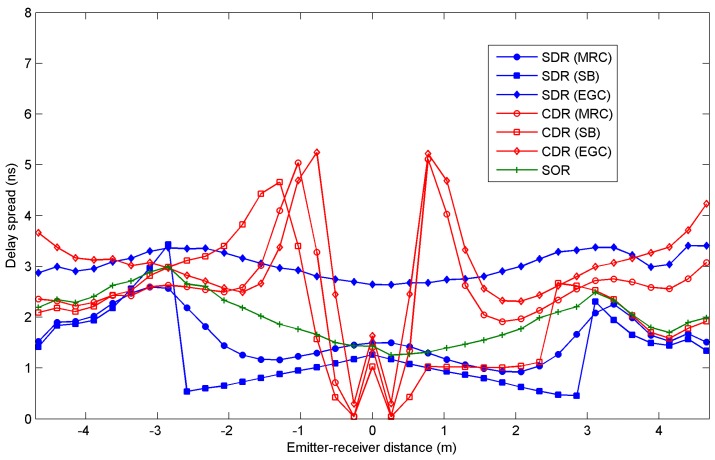
Delay spread for the sectored angle-diversity receiver (SDR), the conventional angle-diversity receiver (CDR) and the self-orienting receiver (SOR) as a function of the emitter-receiver distance along the diagonal from northwest (negative values) to southeast (positive values).

**Figure 6:**
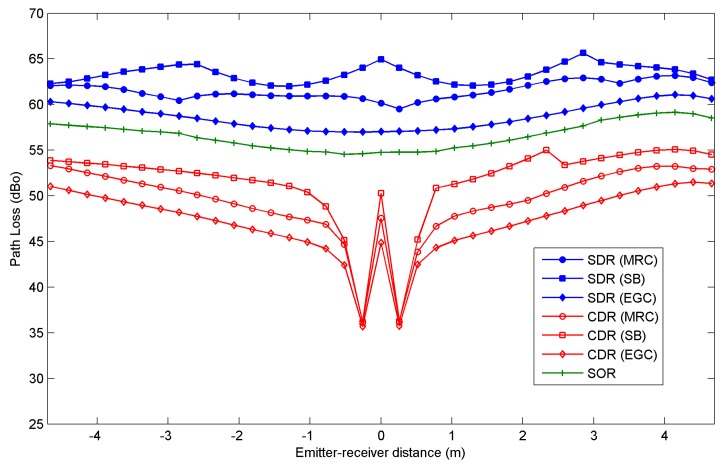
Path loss for the sectored angle-diversity receiver (SDR), the conventional angle-diversity receiver (CDR) and the self-orienting receiver (SOR) as a function of the emitter-receiver distance along the diagonal from northwest (negative values) to southeast (positive values).

Figure 5 and Figure 6 show the rms delay spread and the path loss, respectively, as a function of the distance between the emitter and receiver along a diagonal from the northwest (negative values or distances) to the southeast (positive values or distances) of the room shown in Figure 4. The results were obtained for all three angle-diversity receivers, SDR, CDR, and SOR, when MRC, EGC, and SB combination techniques are applied. In the case of SOR, which only employs a photodetector element, the combining techniques are not applicable. Furthermore, as noted earlier, the SOR receiver employs a maximum search algorithm to aim the receiver in the direction of the highest SNR. In our simulations, in order to find the reception orientation with the best SNR, first the SNR is calculated for all possible reception directions, as defined by their elevation and azimuth angles in steps of its FOV. Secondly, a higher resolution search is carried out around the best SNR obtained in the previous stage, which is bounded by the receiver FOV.

**Figure 7:**
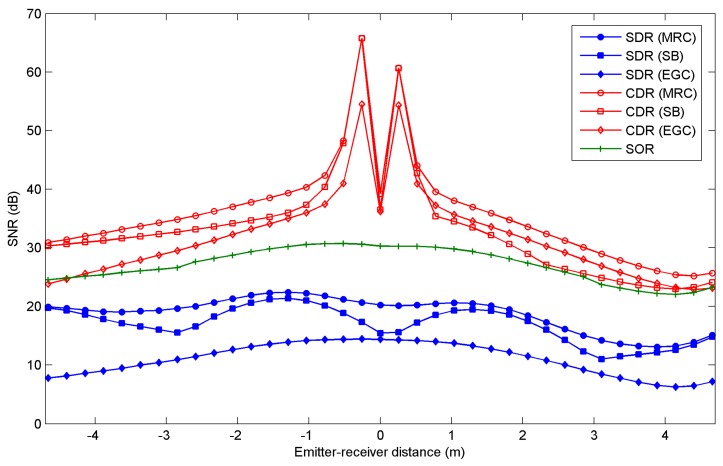
SNR for the sectored angle-diversity receiver (SDR), the conventional angle-diversity receiver (CDR) and the self-orienting receiver (SOR) as a function of the emitter-receiver distance along the northwest- southeast diagonal.

Independently of the angle-diversity receiver employed in the link, all the values obtained for the rms delay spread are below 6 ns, well above the requirements for a receiver operating in a 115 kbps link, i.e., the rms delay spread parameter is not significant when selecting the angle-diversity receiver and the combination technique that provides the best performance. As for the path loss shown in Figure 6, CDR and SOR exhibit smaller values than SDR due to the optical gain provided by the optical front-end employed for the photodetectors in both receivers. The CDR structure exhibits the smallest path loss because its photodetectors have a physical area larger than the SOR photodetector. In term of path loss, the CDR receiver offers the best power efficiency for the power transmitted by the emitter. Specifically, SOR has a path loss about 10 dBo greater than CDR when EGC is used for the emitter located in the centre of the room. The conventional angle-diversity receiver exhibits a unique behaviour when the emitter is located about 0.26 m from the centre of the room. The path loss is minimal because all the radiation is detected by the vertical element of the receiver after undergoing a single reflection. This effect also results in a minimum delay spread since all the radiation reaches the receiver at approximately the same time (see Figure 5), and in a maximum SNR because a low path loss involves a high received power (see Figure 7). In general, the sectored and conventional angle-diversity receivers exhibit the best path loss when EGC is employed, because all the power received by each photodetector element is collected or, put another way, EGC increases the receiver’s total FOV. In MRC, the power received by the elements with a low SNR is attenuated, and in SB, only the power received by the element with the best SNR is taken into account. In short, EGC offers the best path loss if the noise is significant and the signal power is uniformly distributed throughout the room (broadcast communication).

In order to obtain the SNR given by Equation (21), it is necessary to determine the total noise variance as the sum of the contributions from the background light-induced shot noise and thermal noise due to the amplifier. To this end, a bit rate of *R_b_* = 115 kbps was considered since the study involves the application of non-imaging angle-diversity receivers to indoor IR wireless sensor networks. The shot noise can be computed using Equation (23), where the incident optical power from ambient light *P_bg_* originates at the windows and six tungsten bulbs located in the ceiling of the room (see Figure 4). To compute the incident optical power from the windows, each window surface is divided into small square elements of equal area (25 cm^2^), and each element is modelled as a first-order Lambertian emitter with a spectral radiant emittance of 0.20 W/nm/m^2^ [8], i.e., the noise PSD of each element is 0.5 mW/nm. The noise optical power emitted by each element can be obtained by multiplying the noise PSD by *Δλ*, which represents the spectral bandwidth of the optical filter used to limit the ambient radiation reaching the photodetector, which was set at 50 nm for the CDR and SOR structures and 320 nm for SDR. Therefore, applying the Monte Carlo ray-tracing algorithm to each element *E*, and using Equation (8), the incident optical power from the windows act as an ambient light (noise) source that can be expressed as a sum of the form:

(33) $$P_{bg}=\sum_{j=1}^{N_{e}}\left(\sum_{j=0}^{M}h(t;E,R)\right)=\sum_{j=1}^{N_{e}}\left(\frac{1}{d^{2}}R_{E}(\theta ,\;1)A_{eff}(\psi )+\sum_{j=1}^{M-1}p_{k}\right)$$

where *M* = *t_max_*/_Δt_ is the number of time intervals of width *Δt*, and *N_e_* is the number of elements used to divide the window surface. Moreover, the radiant intensity from the bulbs can be modelled as Lambertian sources of second order with an optical spectral density of 0.037 W/nm. Analogously to the calculation of the noise power from ambient light, the incident optical power from the bulb can be obtained by multiplying the power spectral density by *Δλ*. As in the previous case, the optical power contribution from a bulb *E* can be calculated by:

(34) $$P_{bg}=\frac{1}{d^{2}}R_{E}(\theta ,\;2)A_{eff}(\psi )+\sum_{j=1}^{M-1}p_{k}.$$

Assuming typical parameters for a receiver that relies on a FET-based transimpedance preamplifier, i.e., *k* = 1.38 × 10^−23^ J/K, *T* = 295 K, *η* = 175 pF/cm^2^, *R_f_* = 10 kΩ, *g_m_* = 40 mS, *C_g_* = 1 pF, *R_D_* = 146 Ω, *Γ* = 1.5, *K* = 294 fA, *a* = 1, *I_D_* = 20 mA, *I_2_* = 0.562, I_3_ = 0.0868, and *I_f_* = 0.184, Equation (25), which defines the thermal noise, can be expressed just in terms of the physical area of the photodetector. Thus, the thermal noise can be easily computed for each photodetector element of the angle-diversity receiver used in the link.

Figure 7 shows the SNR as function of the emitter-receiver distance along the northwest-southeast diagonal for the three angle-diversity receivers, when MRC, EGC, and SB are applied. In general, CDR and SDR exhibit the highest and the lowest SNR, respectively, with a difference of about 20 dB for the emitter located in the centre of the room. Independently of the receiver, the SNR degrades when the emitter is moved towards the corners of the room, similar to the path loss. Furthermore, when emitters are located along the southeast diagonal of the room, the SNRs are a little lower than along the northwest diagonal. In the southeast corner, the emitters are close to the windows, so the desired signal and the contribution from the shot noise due to the natural ambient light are received of the same direction. In short, the conventional angle-diversity receiver exhibits the best simultaneous SNR and path loss. Furthermore, as expected, for conventional and sectored angle-diversity receivers, the MRC scheme provides the best results, followed by SB and EGC. However, the MRC and SB combining techniques require estimating the SNR at each diversity branch, and the difference between the SNR obtained by MRC and EGC is only about 5 dB for the CDR structure. This is not significant when selecting the receiver and the combining technique that offer the best performance in a link operating at 115 kbps.

Assuming the emitter transmits at 115 kbps using on-off keying (OOK) with non-return-to-zero (NRZ) pulses, the channel is distortionless, the preamplifier is followed by an equalizer that converts the received pulse to one, having a raised-cosine Fourier transform with 100% excess bandwidth, and the equalizer gain is chosen so that when sampled, its output is either 0 or 2*RP_S_* (A), ignoring noise. Each sample of the equalizer output contains Gaussian noise having a total variance that is the sum of contributions from shot and thermal noises. In these conditions [4], the Bit Error Rate (BER) is given by:

(35) BER=Q(SNR), where Q(x)=12π∫x∞e−y22dy

According to Equation (35), to achieve a BER = 10^−9^ requires a SNR = 15.6 dB. Based on the results shown in Figure 7, the CDR and SOR receivers ensure a BER below 10^−9^ for any location along the diagonal of the room. The minimum SNR for SDR is about 10 dB, ensuring a bit error rate below 10^−3^ for a total emitted power of 15 mW. In general, regardless of the combining technique employed, CDR shows a better SNR and path loss than the SDR structure. It is also evident that in terms of both parameters, the CDR receiver provides the best performance. Although the self-orienting receiver exhibits a SNR almost similar to CDR, SOR presents a path loss worse than CDR, regardless of the combining technique applied. Furthermore, SOR needs to implement a SNR estimator, include a search algorithm that automatically aims the receiver towards the highest SNR, and incorporate an electromechanical orienting system. As was previously mentioned, the sensor nodes in a sensor network are limited in power and computational capacity. Therefore, using the SOR as the input sensor for the nodes in an indoor infrared-based sensor network requires additional power consumption and computational capacity over using a CDR in conjunction with EGC. In short, the use of a conventional angle-diversity receiver in conjunction with the EGC technique yields the best trade-off between SNR, computational capacity, and transmitted power. Finally, the use of multi-beam transmitters in conjunction with angle-diversity receivers and power efficient modulation schemes should be analyzed in future research in order to minimize the transmitted power requirements due to the communication between the sensor nodes in an infrared-based sensor network.

## Figures and Tables

**Table 1:** Simulation parameters.

Parameter			Value
Room:	width (*x*), m		6
	length (*y*), m		7.8
	height (*z*), m		2.75
Emitter:	mode (*n*)		1
	Power (*P_E_*), mW		15
	position (*x, y, z*), m		(-, -, 1)
Receivers:	photodetectors: responsivity (*R*), A/W		0.6
	photodetectors: minimum power detected, W		10^−12^
	position (*x, y, z*), m		(3, 3.9, 1)
CPC:	FOV		50°
	refractive index		1.8
	exit aperture, mm		5.64
Bandpass filter:	number of layers		20
	peak transmission (*T_0_*)		0.92
	effective index (*n_s_*)		2.293
	filter order (*m*)		3
	angular bandwidth (∆*ψ*), degrees		50
	spectral bandwidth (∆*λ*), nm		50
	*λ_0_*, nm		810
Longpass filter:	filter transmission (*T_0_*)		0.99
	cutoff wavelength, nm		780
	filter-photodetector combination (∆*λ*), nm		320
Tungsten lamps:	mode (*n*)		2
	lamp power-spectral density, W/nm		0.037
	position (x_1_,y_1_,z_1_), m		(1.5, 1.4, 2.75)
	position (x_2_,y_2_,z_2_), m		(4.5, 1.4, 2.75)
	position (x_3_,y_3_,z_3_), m		(1.5, 3.9, 2.75)
	position (x_4_,y_4_,z_4_), m		(4.5, 3.9, 2.75)
	position (x_5_,y_5_,z_5_), m		(1.5, 6.4, 2.75)
	position (x_6_,y_6_,z_6_), m		(4.5, 6.4, 2.75)
Window:	spectral radiant emittance, W/nm/m^2^		0.2
Resolution:	*∆t*, ns		0.2
Bounces:	*k*		20
Number of rays:	*N*		500,000
Materials	*ρ*	*r_d_*	*m*
Wood	0.63	0.6	3
Varnished W.	0.75	0.3	97
Cement	0.40	1.0	---
Ceramic floor	0.16	0.7	20
Glass	0.03	0.0	280
